# Comment on “Gut Microbiota as a Driver of Inflammation in Nonalcoholic Fatty Liver Disease”

**DOI:** 10.1155/2018/3746509

**Published:** 2018-07-19

**Authors:** Xia Li, Feng-Lai Yuan

**Affiliations:** Department of Orthopaedics and Central Laboratory, The Third Hospital Affiliated to Nantong University, Wuxi, Jiangsu 214041, China

We read with great interest the review article written by Bibbò et al. [1] entitled “Gut Microbiota as a Driver of Inflammation in Nonalcoholic Fatty Liver Disease,” in which the authors reported that intestinal dysbiosis plays a major role in promoting inflammation and progression of nonalcoholic fatty liver disease (NAFLD) to nonalcoholic steatohepatitis (NASH). The authors should be congratulated for this excellent review.

NASH has become one of the most common causes of liver disease in industrialized countries [2] and is characterized by hepatic fatty infiltration, hepatocyte injury, and hepatic inflammation. During the last decade, accumulating evidence supporting the role of gut microbiota in the initiation and progression of NAFLD has been reported by several groups [3]. In addition to the well-documented regulatory mechanism by which the gut microbiota modulate intestinal permeability-induced inflammation, which contributes to the development of NAFLD [1], another mechanism involved in NAFLD development and progression is as follows [4]: (1) gut microbiota modulate host energy metabolism, which causes the accumulation of triglycerides in adipocytes; (2) an alteration in gut microbiota participates in the development of insulin resistance involved in NAFLD pathogenesis by increasing free fatty acids produced by adipocytes; (3) gut microbiota dysbiosis decreases choline metabolism and increases toxic methylamines, which causes the abnormal accumulation of lipids and inflammation in the liver; (4) gut microbiota alter bile acid metabolism, which contributes indirectly to the development of NAFLD; and (5) gut microbiota produce a large number of potentially hepatotoxic compounds, such as ethanol, phenols, and ammonia.

Considering that gut microbiota dysbiosis is a driver of inflammation in the development of NAFLD, the reverse modulation of intestinal dysbiosis may alter the disease process. There is emerging interest in the modulation of gut microbiota to induce benefits in inflammatory intestinal disorders, such as probiotic use, antibiotic treatment, and fecal microbiota transplantation. Although diet can significantly influence the composition of gut microbiota, clinical trials investigating the effects of dietary interventions on the gut microbiota of NAFLD patients are lacking. To ascertain the exact mechanisms of action of gut microbiota in NAFLD, additional human studies with larger patient populations and animal studies are needed. Unraveling the relationship between gut microbiota and the development of NAFLD may then allow for the identification of relevant targets for future therapeutic intervention.
